# Noble Women Workers in the War

**Published:** 1917-04-21

**Authors:** 


### NOBLE WOMEN WORKERS IN THE WAR

The best work and the most zealous workers will not be found continually in the columns of the newspapers. That is the experience of every administrator and man of affairs in exact proportion to the quality and extent of his knowledge and observation. The most popular personage in Great Britain and throughout the Empire is a Lady whom the people love and for whom the sentiment of affection is so deep and genuine that there is not a man or a woman amongst us who would not welcome the opportunity to do her service. H.M. Queen Alexandra has divided her energies and time continuously, untiringly, and with a full heart to the purpose of doing everything possible within her power to help and comfort our wounded warriors, and to provide adequately for the equipment, efficiency, comfort, health, and recognition of the Nursing Services of which she is the illustrious head. We have no official facts to guide us, but we are confident from our observation that it is the simple truth that Queen Alexandra has not failed to receive at Marlborough House a single matron, sister, or staff nurse of the Naval and Military Nursing Services who has been awarded the Royal Red Cross or been received by His Majesty the King at Buckingham Palace. How many other workers, officials, and warriors, Queen Alexandra has done her utmost to cheer and help, encourage and entertain, cannot be stated. Her Majesty's work is done so quietly and out of the limelight. Let it suffice that Queen Alexandra is the kindest-hearted and most generous woman who has ever worn a crown. To know and serve her is an education in character, and a revelation of the high standard which the Greatest in the Land attain to in these strenuous and critical times of Empire.

Field-Marshal Viscount French did good service on Sunday in Hull, when he paid a warm and deserved tribute to the war work of the women of the Empire. He has had exceptional opportunities of being taken all over the country on duty, and has so gained a greater insight than most people into women's work, and that work not confined to one class but embracing women of all classes and all ages who have splendidly joined in. He has seen in the munition works young girls doing the work of strong men, not only willingly, but doing it as though they liked it. In Lord French's view *the most self-sacrificing work of all by women is*, however, *that of the hospital*. Lord French declared the services of our women will form a brilliant page when the history of the war comes to be written.

We have had opportunities, too, of assessing and judging the variety and quality of work done by women in wartime in the hardest field of all, the hospital, where not a few matrons of great hospitals have taken on the responsible extra and onerous duty of Principal Matron, involving sometimes the organisation, preparation and equipment, and always the oversight and much responsibility for the administration of buildings, new, converted, or adapted for hospital purposes and the reception of wounded warriors. The strain and responsibility have so told on some of these zealous and able workers in the Homeland, that we have felt it a duty to try and induce the worker to relax a little, and get away for a rest and change. But the spirit of the woman always rebelled, for she was doing her work in the war, and that work in her view must be pursued continuously, with all her energies, until the war was ended and done with. Another notable fact is that in the offices of the Matrons-in-Chief some very able and devoted women, being hospital matrons, have worked in the background with an industry and ability that deserve the highest commendation. They may have been disregarded and unnoticed except by their immediate chief, but they have continued to serve with zeal and devotion, and have taken a most important part in the successful organisation and perfecting of many things and the great results which have followed. How many of these ladies are there, still, who have not received a decoration nor any honourable mention from the fount of honour?

Next there are the sisters and charge nurses who have taken the place of other sisters and charge nurses attacked by the war fever, which concentrates the energies of its victims on getting out of the country into the field of war. We are of opinion, and it is justly held by the most knowledgeable and appreciative observers of really good service by women and others, that of all war workers the ones whose interests should be most tenderly looked after from the centre and by the authorities are those who have been content to keep out of the limelight, to stick to the drudgery and strain of work in the Homeland, whilst they steadily pursue the arduous duties left them by their restless sisters, who, in common parlance, have been "*called* to the Front." We have confidence that these Homeland workers, wherever their work may lie, however far it may be from the point of publicity and the prying eyes of the photographer, will one day have good cause to be satisfied with the recognition that they will earn. The nation owes all women workers more than words can adequately express, but of them all the types we have mentioned should be made a note of by those in high places, as well as by the hospital authorities

For Matrons' and Sisters' Appointments, see p. 58.

under whom they are working, and care should be taken that every one of them may be specially honoured by the representatives of a grateful nation and Empire.

Our readers will find on page 49 some interesting reminiscences of nursing, its evolution and early difficulties. They will there find, too, an indication of the days when the nurses' sleeping accommodation was in the basement, in rooms with stone floors, in contrast with the higher class of nurses' accommodation now insisted on at the best modern hospitals, where it is worthy of the best nurses and the value which ought to attach to the maintenance of their health, the preservation of their spirit, the elevation of their training, and the gift to them of a good share of the pleasures of life.

As we go to press we are informed that the Queen has given her patronage to a concert for nurses which is being arranged by Mr. Victor Bergel, with the assistance of a number of people who are interested in hospital work. The concert will take place at the Queen's Hall, Langham Place, on Wednesday, May 2, at 3 P.M., and will be under the auspices of the College of Nursing. Admission will be by invitation only. Application for tickets should be made to the Secretary, College of Nursing, 6 Vere Street, W.

